# Positive Association Between Serum Alkaline Phosphatase and First Stroke in Hypertensive Adults

**DOI:** 10.3389/fcvm.2021.749196

**Published:** 2021-12-10

**Authors:** Yuanyuan Zhang, Huan Li, Di Xie, Jianping Li, Yan Zhang, Binyan Wang, Chengzhang Liu, Yun Song, Xiaobin Wang, Yong Huo, Fan Fan Hou, Xiping Xu, Xianhui Qin

**Affiliations:** ^1^Division of Nephrology, Nanfang Hospital, Southern Medical University, Guangzhou, China; ^2^National Clinical Research Center for Kidney Disease, Guangzhou, China; ^3^State Key Laboratory of Organ Failure Research, Guangzhou, China; ^4^Guangdong Provincial Institute of Nephrology, Guangzhou, China; ^5^Guangdong Provincial Clinical Research Center for Kidney Disease, Guangzhou, China; ^6^Guangdong Provincial Key Laboratory of Renal Failure Research, Guangzhou, China; ^7^Guangzhou Regenerative Medicine and Health Guangdong Laboratory, Guangzhou, China; ^8^Department of Cardiology, Peking University First Hospital, Beijing, China; ^9^Institute of Biomedicine, Anhui Medical University, Hefei, China; ^10^Beijing Advanced Innovation Center for Food Nutrition and Human Health, College of Food Science and Nutritional Engineering, China Agricultural University, Beijing, China; ^11^Department of Population, Family and Reproductive Health, Johns Hopkins University Bloomberg School of Public Health, Baltimore, MD, United States

**Keywords:** alkaline phosphatase, first ischemic stroke, first stroke, hypertension, cohort study

## Abstract

The relation of alkaline phosphatase (ALP) with stroke risk remains uncertain. We aimed to examine the association between serum ALP and the risk of first stroke, and explore the possible effect modifiers in the association, among adults with hypertension. A total of 19,747 participants with baseline ALP measurements and without liver disease at baseline from the China Stroke Primary Prevention Trial (CSPPT) were included. The primary outcome was a first stroke. Over a median follow-up of 4.5 years, there was a positive association between serum ALP levels and the risk of first stroke (per SD increment, adjusted HR, 1.10; 95%CI: 1.01, 1.20). When serum ALP was evaluated as quartiles, a significantly higher risk of first stroke was observed in those in quartile 2–4 (ALP ≥79 IU/L; adjusted HR, 1.38; 95% CI: 1.11, 1.71), compared with participants in quartile 1 (ALP <79 IU/L). Similar results were found for first ischemic or hemorrhagic stroke. Similar findings were also found in those with a normal range of baseline ALP levels (20–140 IU/L) (per SD increment, adjusted HR, 1.15; 95%CI: 1.05, 1.27). None of the variables, including sex, age, body mass index, smoking, alcohol drinking, blood pressure, total cholesterol, fasting glucose levels at baseline, and blood pressure levels during the treatment period, significantly modified the association. In summary, our study suggests that higher serum ALP levels, even in normal range, were significantly related to higher risk of first stroke among Chinese hypertensive adults.

## Introduction

Stroke has become a worldwide public health concern worldwide (1, 2). Previous studies had reported that traditional risk factors could not explain all first stroke risk (3–5). Therefore, it is important to identify more risk factors to further lower residual risk of first stroke.

Alkaline phosphatase (ALP) is a clinical marker of bone or hepatic disease (6). Previous studies have found that higher ALP was related to vascular calcification by catalyzing the hydrolysis of organic pyrophosphate, which is an inhibitor of vascular calcification (6). This indicted that ALP may have an important role in the development of vascular disease. Accordingly, previous studies showed that higher serum ALP was related to higher cardiovascular disease (CVD) (7–9), and cerebral small vessel disease (10–12). However, data from prospective studies on the relation of ALP with stroke risk are limited and inconclusive (8, 9, 13). Moreover, few previous studies have fully examined the potential effect modifiers on the relationship of ALP with the risk of first stroke.

Hypertension has been shown to be one of the most important modifiable risk factors of stroke (14, 15). Therefore, using date from the China Stroke Primary Prevention Trial (CSPPT) (16), we aimed to investigate the association between serum ALP and the risk of first stroke and examine the possible effect modifiers for the association in adults with hypertension.

## Materials and Methods

### Study Design and Participants

The detailed methods and major findings of the CSPPT (ClinicalTrials.gov identifier NCT00794885) have been reported previously elsewhere. (16–19) Briefly, the CSPPT was a randomized, double-blind, controlled trial conducted in 20,702 45–75 years adults with hypertension from May 19, 2008 to August 24, 2013 in 32 communities in Anhui and Jiangsu provinces of China.

Our current study, as a *post-hoc* analysis of the CSPPT, included a total of 19,747 participants with complete data on ALP measurements and without liver disease, including self-reported chronic hepatitis, hepatic adipose infiltration, or cirrhosis at baseline (Supplementary Figure 1).

The CSPPT and the current study were approved by the Ethics Committee of the Institute of Biomedicine, Anhui Medical University, Hefei, China (FWA assurance number: FWA00001263). All participants provided written informed consent.

### Intervention and Follow-Up

In the CSPPT, eligible participants were randomly assigned, in a 1:1 ratio, to one of two treatment groups: a daily oral dose of one tablet containing 10 mg enalapril and 0.8 mg folic acid (the enalapril-folic acid group), or one tablet containing 10 mg enalapril only (the enalapril-only group).

Participants were followed up every 3 months. BP was measured; usage of concomitant medications, adverse events, study drug compliance and possible endpoint events were documented at each follow-up visit.

### Laboratory Assessment

Serum concentrations of fasting ALP, alanine aminotransferase (ALT), gamma glutamyl transpeptidase (GGT), total bilirubin (TBIL), aspartate aminotransferase (AST), total homocysteine (tHcy), lipids, creatinine, glucose, albumin, and fasting glucose were measured using automatic clinical analyzers (Beckman Coulter) at the core laboratory of the National Clinical Research Center for Kidney Disease, Nanfang Hospital, Guangzhou, China.

### Study Outcomes

The primary outcome was a first, nonfatal or fatal stroke, excluding subarachnoid hemorrhage and silent stroke. Secondary outcomes included a first ischemic stroke and a first hemorrhagic stroke. The study outcomes were adjudicated by an independent Endpoint Adjudication Committee, whose members were unaware of treatments assignments (16).

### Statistical Analysis

Means ± standard deviations (SDs) or medians [interquartile range (IQR)] for continuous variables and proportions for categorical variables, were calculated for population characteristics by baseline serum ALP quartiles (<79 IU/L, 79 to <96 IU/L, 96 to <118 IU/L, ≥118 IU/L). Differences in baseline characteristics was compared using ANOVA tests or chi-square tests, accordingly.

The association between baseline serum ALP and the risk of first stroke were estimated with the use of Cox proportional hazards models [hazards ratio (HR) and 95% confidence interval (CI)] without and with adjustments for study centers, treatment groups, age, sex, systolic blood pressure (SBP), smoking, body mass index, alcohol drinking, fasting glucose, total cholesterol, triglyceride, albumin, creatinine, tHcy, methylenetetrahydrofolate reductase (*MTHFR*) C677T genotypes and antihypertensive treatment at baseline, as well as time-averaged SBP during the follow up. Moreover, the possible effect modifiers in the association between serum ALP and the risk of stroke were evaluated by stratified analyses and their interactions were assessed.

A two-tailed *P* < 0.05 was considered statistically significant in all the analyses. R software, version 3.6.1 (http://www.R-project.org/) was used for all analyses.

## Results

### Study Participants and Baseline Characteristics

As illustrated in the flow chart (Supplementary Figure 1), in the current study, 19,747 participants with baseline ALP measurements and without baseline liver disease, were included.

The median and mean serum ALP levels were 96 IU/L and 100.7 IU/L (SD, 31.2), respectively. Characteristics of participants by ALP quartiles are presented in Table 1. Participants with higher ALP levels were older and more likely to be female; had higher HDL-C, TG, FG, SBP, albumin, folate at baseline and time-averaged SBP levels during the follow-up; had lower DBP, BMI, total cholesterol, tHcy, creatinine at baseline and time-averaged DBP levels during the follow up; and had lower frequency in the antiplatelet and antihypertensive drugs usage, as wells as lower frequency in C677 TT genotype, current smoking, alcohol drinking consumptions at baseline (Table 1).

**Table 1:** Characteristics of the study participants by baseline serum alkaline phosphatase quartiles.

**Variables^a^**	**Baseline serum alkaline phosphatase quartiles, IU/L**				***P*-value**
	**Q1 (<79)**	**Q2 (79– <96)**	**Q3 (96– <118)**	**Q4 (≥118)**	
*N*	4,695	49,19	5,182	4,951	
Male, No. (%)	2,422 (51.6)	2,235 (45.4)	1,987 (38.3)	1,390 (28.1)	<0.001
Age, year	58.3 ± 8.2	59.7 ± 7.6	60.6 ± 7.2	61.3 ± 6.8	<0.001
Body mass index, kg/m^2^	25.4 ± 3.6	25.1 ± 3.6	24.9 ± 3.7	24.5 ± 3.7	<0.001
Current smoking, No. (%)	1324 (28.2)	1269 (25.8)	1152 (22.2)	866 (17.5)	<0.001
Current alcohol drinking, No. (%)	1576 (33.6)	1291 (26.2)	1122 (21.7)	750 (15.2)	<0.001
*MTHFR* 677 TT, No. (%)	1205 (25.7)	1210 (24.6)	1198 (23.1)	1102 (22.3)	<0.001
Enalapril-folic acid, No. (%)	2375 (50.6)	2454 (49.9)	2561 (49.4)	2493 (50.4)	0.661
BP, mmHg					
SBP at baseline	165.6 ± 20.6	167.3 ± 20.5	167.4 ± 20.6	168.2 ± 20.3	<0.001
DBP at baseline	95.3 ± 12.0	94.5 ± 12.0	94.1 ± 11.7	93.1 ± 11.9	<0.001
Time-averaged on-treatment SBP	139.3 ± 11.3	140.0 ± 11.2	139.7 ± 11.4	140.5 ± 11.9	<0.001
Time-averaged on-treatment DBP	84.3 ± 7.5	83.5 ± 7.7	82.8 ± 7.4	82.0 ± 7.8	<0.001
Laboratory results					
Total cholesterol, mmol/L	5.6 ± 1.2	5.6 ± 1.2	5.5 ± 1.2	5.4 ± 1.2	<0.001
HDL-C, mmol/L	1.3 ± 0.4	1.3 ± 0.4	1.3 ± 0.4	1.4 ± 0.4	<0.001
Triglycerides, mmol/L	1.6 ± 1.7	1.6 ± 1.0	1.7 ± 0.9	1.8 ± 1.0	<0.001
Fasting glucose, mmol/L	5.8 ± 1.4	5.8 ± 1.5	5.8 ± 1.7	5.9 ± 2.1	0.034
Creatinine, μmol/L	68.6 ± 16.5	66.7 ± 17.2	65.2 ± 16.9	63.4 ± 25.2	<0.001
Folate, ng/mL	7.9 ± 3.3	8.3 ± 3.8	8.6 ± 3.9	9.4 ± 4.4	<0.001
Total homocysteine, μmol/L	14.9 ± 10.0	14.5 ± 8.6	14.5 ± 8.1	14.1 ± 7.0	<0.001
Albumin, g/L	47.8 ± 5.6	48.8 ± 4.9	49.4 ± 5.5	50.3 ± 6.9	<0.001
Alkaline phosphatase, IU/L	65.9 ± 10.4	87.1 ± 4.8	105.6 ± 6.3	142.3 ± 24.8	<0.001
Alanine transaminase, IU/L	11.0 (9.0, 15.0)	12.0 (9.0, 16.0)	13.0 (10.0, 17.0)	14.0 (11.0, 19.6)	<0.001
Aspartate transaminase, IU/L	21.4 (18.0, 26.1)	22.8 (19.2, 27.8)	24.0 (19.9, 29.5)	26.6 (21.6, 33.4)	<0.001
Gamma glutamyl transpeptidase, IU/L	19.6 (14.5, 28.7)	19.8 (14.8, 28.5)	19.9 (14.9, 29.4)	20.5 (15.2, 30.2)	<0.001
Medication use, No. (%)					
Antihypertensive drugs	2,399 (51.1)	2,315 (47.1)	2,371 (45.8)	2,062 (41.6)	<0.001
Glucose-lowering drugs	59 (1.3)	84 (1.7)	75 (1.4)	86 (1.7)	0.175
Lipid-lowering drugs	46 (1.0)	43 (0.9)	32 (0.6)	35 (0.7)	0.171
Antiplatelet drugs	196 (4.2)	164 (3.3)	143 (2.8)	92 (1.9)	<0.001

a *Continuous variables are presented as Mean ± SD or IQR (25^th^, 75^th^), categorical variables are presented as n (%)*.

*BP, blood pressure; DBP, diastolic blood pressure; HDL-C, high-density lipoprotein; MTHFR, methylenetetrahydrofolate reductase; SBP, systolic blood pressure*.

In addition, those with higher ALP levels had a lower frequency usage of diuretics and a higher frequency usage of glucose-lowering drugs during the follow up (Supplementary Table 1).

### Baseline Serum ALP and the Risk of First Stroke

We first evaluated the association of ALP with other covariates at baseline, and found that age, sex, BMI, SBP, current smoking, alcohol drinking, total cholesterol, triglycerides, fasting glucose, tHcy, creatinine, albumin and time-averaged SBP levels were significantly associated with ALP levels (all *P* < 0.05) (Supplementary Table 2). As such, we included all above covariates in the regression models for the association between serum ALP and the risk of first stroke.

During the median follow-up duration of 4.5 years (25^th^-75^th^ percentile, 4.2–4.7), there was a significant positive relation of serum ALP with first total stroke (per SD increment, adjusted HR, 1.10; 95%CI: 1.01, 1.20) (Table 2). When serum ALP was evaluated as quartiles, the adjusted HRs (95%CI) were 1.00 (ref.), 1.28 (1.00, 1.64), 1.44 (1.13, 1.85), 1.47 (1.13, 1.93), according to quartile 1 (<79 IU/L), quartile 2 (79 to <96 IU/L), quartile 3 (96 to <118 IU/L), quartile 4 (≥118 IU/L), respectively. Consistently, a higher risk of first total stroke was observed in those in quartile 2-4 (ALP ≥79 IU/L; 3.2% *vs*. 2.6%; adjusted HR, 1.38; 95% CI: 1.11, 1.71), compared with participants in quartile 1 (ALP <79 IU/L) (Table 2). Similar trends were observed for first ischemic stroke (ALP ≥79 *vs*. <79 IU/L; adjusted HR, 1.37; 95% CI: 1.08, 1.73), and first hemorrhagic stroke (ALP ≥79 *vs*. <79 IU/L; adjusted HR, 1.57; 95% CI: 0.91, 2.72) (Table 2).

**Table 2:** The association between baseline alkaline phosphatase and the risk of first stroke.

**ALP, IU/L**	**N**	**No. of events (%)**	**Crude model**		**Adjusted model^a^**	
			**HR (95% CI)**	***P*-value**	**HR (95% CI)**	***P*-value**
First total stroke						
Continuous, per SD increment	19,747	606 (3.1)	1.04 (0.96, 1.13)	0.311	1.10 (1.01, 1.20)	0.036
Quartiles						
Q1 (<79)	4,695	123 (2.6)	1.00		1.00	
Q2 (79– <96)	4,919	154 (3.1)	1.20 (0.95, 1.52)	0.133	1.28 (1.00, 1.64)	0.047
Q3 (96– <118)	5,182	172 (3.3)	1.26 (1.00, 1.59)	0.049	1.44 (1.13, 1.85)	0.004
Q4 (≥118)	4,951	157 (3.2)	1.21 (0.95, 1.53)	0.116	1.47 (1.13, 1.93)	0.004
*P* for trend			0.114		0.003	
Categories						
Q1 (<79)	4,695	123 (2.6)	1.00		1.00	
Q2–4 (≥79)	15,052	483 (3.2)	1.22 (1.00, 1.49)	0.046	1.38 (1.11, 1.71)	0.003
First ischemic stroke						
Continuous, per SD increment	19,747	493 (2.5)	1.04 (0.95, 1.13)	0.369	1.12 (1.02, 1.24)	0.020
Categories						
Q1 (<79)	4,695	101 (2.2)	1.00		1.00	
Q2–4 (≥79)	15,052	392 (2.6)	1.21 (0.97, 1.51)	0.088	1.37 (1.08, 1.73)	0.010
First hemorrhagic stroke						
Continuous, per SD increment	19,747	111 (0.6)	1.05 (0.88, 1.26)	0.577	1.05 (0.85, 1.29)	0.644
Categories						
Q1 (<79)	4,695	21 (0.4)	1.00		1.00	
Q2–4 (≥79)	15,052	90 (0.6)	1.33 (0.83, 2.14)	0.239	1.57 (0.91, 2.72)	0.103

a *Adjusted for study centers, treatment groups, age, sex, body mass index, smoking, alcohol drinking, systolic blood pressure (SBP), albumin, total cholesterol, triglyceride, fasting glucose, creatinine, total homocysteine, methylenetetrahydrofolate reductase (MTHFR) C677T genotypes and antihypertensive treatment at baseline, as well as time-averaged SBP during the treatment period*.

*ALP, alkaline phosphatase*.

Moreover, similar findings were also observed in those with a normal range of serum ALP (20-140 IU/L) (20) levels (per SD increment, adjusted HR, 1.15; 95%CI: 1.05, 1.27) (Supplementary Table 3). More importantly, further adjustments for diuretics, calcium channel blockers, and glucose-lowering drugs during the follow-up (ALP ≥79 *vs*. <79 IU/L; adjusted HR, 1.38; 95%CI: 1.12, 1.72) (Supplementary Table 4), or other liver enzymes, including AST, ALT and GGT (ALP ≥79 *vs*. <79 IU/L; adjusted HR, 1.40; 95% CI: 1.12, 1.75) (Supplementary Table 5) did not materially alter the findings.

### Stratified Analyses

Considering the substantial ALP difference between men and women, we investigated the ALP-stroke association stratified by sex. In males, compared to participants with ALP <79 IU/L, a higher risk of first stroke was observed in those in quartile 2–4 (ALP ≥79 IU/L; adjusted HR, 1.38; 95%CI: 1.04, 1.82). Similar results were found in females (adjusted HR, 1.39; 95%CI: 1.00, 1.91; *P* for interaction = 0.979) (Table 3).

**Table 3:** The association between baseline alkaline phosphatase and the risk of first stroke in various subgroups.

**Subgroups**	**N**	**Q1 (<79)** **Events (%)**	**Q2-4 (≥79) Events (%)**	**Adjusted HR** (95%CI) *	***P*-interaction^a^**
Sex					0.979
Male	8,034	73 (3.0)	212 (3.8)	1.38 (1.04, 1.82)	
Female	11,713	50 (2.2)	271 (2.9)	1.39 (1.00, 1.91)	
Age, year					0.143
<60	10,000	47 (1.7)	186 (2.6)	1.69 (1.21, 2.36)	
≥60	9,747	76 (3.9)	297 (3.8)	1.24 (0.94, 1.62)	
BMI, kg/m^2^					0.250
<24	8,087	47 (2.8)	185 (2.9)	1.18 (0.84, 1.65)	
≥24	11,653	76 (2.5)	298 (3.4)	1.51 (1.15, 1.98)	
Treatment group					0.597
Enalapril	9,864	66 (2.8)	272 (3.6)	1.45 (1.09, 1.93)	
Enalapril-folic acid	9,883	57 (2.4)	211 (2.8)	1.30 (0.95, 1.77)	
Current smoking					0.381
No	15,135	75 (2.2)	352 (3.0)	1.48 (1.13, 1.95)	
Yes	4,611	48 (3.6)	131 (4.0)	1.23 (0.87, 1.72)	
Current Alcohol drinking				0.340	
No	15,004	71 (2.3)	376 (3.2)	1.49 (1.14, 1.96)	
Yes	4,739	52 (3.3)	107 (3.4)	1.21 (0.86, 1.70)	
SBP at baseline, mmHg				0.296	
<160	7,389	26 (1.4)	108 (2.0)	1.69 (1.08, 2.64)	
≥160	12,358	97 (3.5)	375 (3.9)	1.30 (1.02, 1.65)	
Time-averaged SBP during the treatment period, mmHg			0.974		
<140	10,884	38 (1.4)	142 (1.7)	1.38 (0.96, 1.99)	
≥140	8,862	85 (4.3)	341 (4.9)	1.39 (1.08, 1.80)	
Total cholesterol, mmol/L				0.577	
<6.2	14,389	81 (2.4)	313 (2.8)	1.32 (1.02, 1.71)	
≥6.2	5,114	36 (2.8)	160 (4.2)	1.50 (1.03, 2.16)	
Fasting glucose, mmol/L				0.948	
<6.1	14,526	78 (2.3)	321 (2.9)	1.40 (1.08,1.81)	
6.1– <7.0	2,765	21 (2.8)	60 (3.0)	1.28 (0.76,2.14)	
Diabetes	2,221	19 (4.0)	92 (5.3)	1.41 (0.85,2.36)	
Total homocysteine, μmol/L				0.690	
<12.5	9,786	45 (1.9)	201 (2.7)	1.45 (1.04, 2.04)	
≥12.5	9,809	73 (3.1)	276 (3.7)	1.33 (1.02, 1.75)	
Albumin, g/L					0.566
<48.3 (median)	9,811	81 (3.1)	257 (3.6)	1.31 (1.00, 1.71)	
≥48.3	9,931	42 (2.1)	226 (2.9)	1.48 (1.05, 2.10)	
Calcium channel blockers usage during the treatment period		0.787			
No	4,114	18 (1.7)	77 (2.5)	1.48 (0.87, 2.51)	
Yes	15,633	105 (2.9)	406 (3.4)	1.36 (1.08, 1.72)	
Diuretics usage during the treatment period		0.817			
No	9,978	52 (2.4)	213 (2.7)	1.35 (0.97, 1.87)	
Yes	9,769	71 (2.8)	270 (3.7)	1.42 (1.07, 1.87)	

a *If not stratified, adjusted for study centers, treatment groups, age, sex, body mass index, smoking, alcohol drinking, systolic blood pressure (SBP), albumin, total cholesterol, triglyceride, fasting glucose, creatinine, total homocysteine, methylenetetrahydrofolate reductase (MTHFR) C677T genotypes and antihypertensive treatment at baseline, as well as time-averaged SBP during the treatment period*.

*BMI, body mass index*.

Other variables, including treatment group (enalapril vs. enalapril-folic acid), age (≥60 vs. <60 years), BMI (≥24 vs. <24 kg/m^2^), current smoking (yes vs. no), current alcohol drinking (yes vs. no), SBP (<160 vs. ≥160 mmHg), total cholesterol (<6.2 vs. ≥6.2 mmol/L), fasting glucose (<6.1 vs. 6.1– <7.0 mmol/L vs. diabetes), tHcy [ <12.5 (median) vs. ≥12.5 μmol/L], albumin [ <48.3 (median) vs. ≥48.3 g/L] at baseline, as well as time-averaged SBP (≥140 vs. <140 mmHg), diuretics usage (yes vs. no), calcium channel blockers usage (yes vs. no) and over during the follow-up period, also did not significantly modified the association between baseline serum ALP and first total stroke (all *P*-interactions > 0.05) (Table 3).

### Sensitivity Analysis

We first explored the effects of folic acid treatment on ALP levels, and found that there was no significant difference of ALP levels between the 2 treatment groups (enalapril-folic acid group vs. enalapril only group; *P* = 0.669) (Supplementary Table 6). Furthermore, we also did not find the substantial difference in the efficacy of folic acid treatment in prevention of first stroke among participants with different ALP levels (<79 vs. ≥79 IU/L; *P*-interaction = 0.597) (Supplementary Table 7).

## Discussion

Our study first found that among hypertensive adults, those participants with higher serum ALP, even in normal range, had a significantly higher risk of first total stroke. This study has adjusted for a comprehensive range of covariables/ confounders, and multiple subgroup analyses and sensitivity analyses were conducted to ensure the robustness of the study findings.

The association between serum ALP and stroke have been reported by previous cohort studies, but the results have been inconclusive. A recent prospective study, including 2,578 Iranian participants without prevalent CVD at baseline (mean ALP levels: 210.5 IU/L), showed that per one SD increase in ALP level was related to higher risk of stroke (adjusted HR, 1.20; 95%CI: 0.97, 1.49) (8). However, another study reported that there was no obvious relationship between serum ALP and stroke in 3,381 men, aged 60 to 79 years, without a history of MI or stroke at baseline (9). In addition, Shimizu Y et al. (13) reported that for nondrinkers, compared with participants in quintile 3 of ALP levels, higher ALP levels were related to higher risks of ischemic stroke for men and hemorrhagic stroke for women. However, lower ALP levels were related to higher risks of ischemic and hemorrhagic strokes in both men and women among 10,754 Japanese subjects (13). Overall, these studies showed that the relation of ALP and the risk of stroke is still uncertain. Of note, only part of the participants had hypertension in the previous studies. More importantly, there was no information about BP levels during the follow-up period in all of the previous studies. Therefore, the results cannot be generalized to adults with hypertension. Our study provided an opportunity to examine the dose-response association between circulating ALP and first stroke in hypertensive adults receiving standard antihypertensive treatments.

In contrast to the prior studies, our study provides some novel findings. It is by far the first and largest study of its kind demonstrating a positive relation between serum ALP and first stroke during a follow-up period of 4.5 years in general hypertensive adults. This significant association remained with adjustments for comprehensive covariables, including sex, age, BP, BMI, *MTHFR* C677T, smoking, alcohol drinking, lipids, FG, creatinine, albumin, tHcy and antihypertensive drug usage at baseline, and the time averaged BP during the follow-up period. Moreover, we have fully explored possible effect modifiers on the serum ALP-stroke association in general hypertensive adults, the findings suggested that the association between serum ALP and first stroke was consistent in these subgroups.

The exact mechanisms by which higher serum ALP was related to higher risk of stroke remains to be delineated. However, our study's findings are biologically plausible based on the available evidence. First, higher ALP has been reported to be related to vascular calcification by increased bone metabolism (7, 11). An animal experiment in the mouse had found a positive association of serum total ALP activity with bone-type ALP activity in calcified vascular lesions (21). Second, impaired vascular homeostasis may be another possible mechanism. Hematopoietic stem cells derived from bone marrow have an important role in vascular homeostasis (22, 23). Previous studies have reported that osteoblasts, whose activity was positively associated with bone-type ALP expression (24), may regulate the production of hematopoietic stem cells in bone marrow (25–27). As such, serum ALP level may be associated with vascular homeostatic activity. In addition, hematopoietic stem cells play a major role in the pathogenesis of atherosclerosis and may promote angiogenesis (22). Thus, higher ALP might constitute a risk factor for stroke owing to the progressive atherosclerosis. Furthermore, a previous study conducted in hypertensive individuals revealed that higher serum ALP increased the risk of endothelial dysfunction (28). Some possible mechanisms for this may be that ALP was related to reduced nitric oxide (NO) bioavailability by inhibiting tyrosine kinase activity into endothelial cells (29, 30). Taken together, we hypothesized that vascular calcification, atherosclerosis, and endothelial dysfunction associated with higher serum ALP may all be involved in the pathogenesis of first stroke. However, the detailed underlying mechanisms still need to be further examined in future studies.

Limitations of the current study should also be noted. First, our study is a *post-hoc* analysis of the CSPPT. Although a broad array of covariates had been adjusted in the regression models, the residual confounding remains possible. Second, our study was conducted in Chinese adults with hypertension. Caution is needed when generalizing to other populations. Third, the small events of hemorrhagic stroke limited the statistical power of our analysis. Fourth, in our current study, most of the participants had normal ALP levels. Therefore, we could not investigate the possible increased stroke risk associated with relatively very low ALP levels. Fifth, in the present study, we measured total serum ALP rather than ALP isozymes. Finally, the ALP levels were assessed only at baseline and the exit visit. More frequent assays of ALP levels would allow for a more accurate assessment of its progression over time. Therefore, our current study could not provide a causal relationship between ALP and an increased risk of stroke. Overall, our results were just hypothesis generating. All findings need to be further confirmed in more studies.

In summary, our study suggests that higher serum ALP levels, even in normal range, were significantly related to higher risk of first stroke in general hypertensive adults. If our findings were further confirmed, identifying hypertensive patients with higher serum ALP levels may help detect those individuals who are at high risk of first stroke.

## Data Availability Statement

The raw data supporting the conclusions of this article will be made available by the authors, without undue reservation.

## Ethics Statement

The studies involving human participants were reviewed and approved by Ethics Committee of the Institute of Biomedicine, Anhui Medical University, Hefei, China (FWA assurance number: FWA00001263). The patients/participants provided their written informed consent to participate in this study.

## Author Contributions

YuZ, XX, and XQ designed research. YuZ, JL, YaZ, BW, YH, FH, XX, and XQ conducted research. YuZ, HL, DX, JL, YaZ, BW, CL, YS, XW, YH, FH, XX, and XQ contributed to acquisition, analysis, or interpretation of data. YuZ, HL, and XQ performed statistical analysis. YuZ and XQ wrote the paper. All authors revised the manuscript and approved the final version of the manuscript.

## Funding

The study was supported by funding from the following: the National Key Research and Development Program (2016YFE0205400, 2018ZX09739010, 2018ZX09301034003; XX), the Science and Technology Planning Project of Guangzhou, China (201707020010; XX); the Science, Technology and Innovation Committee of Shenzhen (JSGG20170412155639040, GJHS20170314114526143, JSGG20180703155802047; XX); the Economic, Trade and Information Commission of Shenzhen Municipality (20170505161556110, 20170505160926390; XX); the National Natural Science Foundation of China (81730019, 81973133; XQ) and Outstanding Youths Development Scheme of Nanfang Hospital, Southern Medical University (2017J009, XQ). The funders had no role in the design and/or conduct of the study; the preparation, review, or approval of the manuscript; or the decision to submit the manuscript for publication.

## Conflict of Interest

The authors declare that the research was conducted in the absence of any commercial or financial relationships that could be construed as a potential conflict of interest.

## Publisher's Note

All claims expressed in this article are solely those of the authors and do not necessarily represent those of their affiliated organizations, or those of the publisher, the editors and the reviewers. Any product that may be evaluated in this article, or claim that may be made by its manufacturer, is not guaranteed or endorsed by the publisher.

## References

[1] GBD 2016 Causes of Death Collaborators. Global, regional, and national age-sex specific mortality for 264 causes of death, 1980-2016: a systematic analysis for the Global Burden of Disease Study 2016. Lancet. (2017). 390:1151–210. 10.1016/S0140-6736(17)32152-9 28919116 PMC5605883

[2] Benjamin EJ Virani SS Callaway CW Chamberlain AM Chang AR Cheng S. Heart disease and stroke statistics-2018 update: a report from the American Heart Association. Circulation. (2018) 137:e67–492. 10.1161/CIR.0000000000000573 29386200

[3] O'Donnell MJ Chin SL Rangarajan S Xavier D Liu L Zhang H. Global and regional effects of potentially modifiable risk factors associated with acute stroke in 32 countries (INTERSTROKE): a case-control study. Lancet. (2016) 388:761–75. 10.1016/S0140-6736(16)30506-2 27431356

[4] Qin X Li J Cui Y Liu Z Zhao Z Ge J. Effect of folic acid intervention on the change of serum folate level in hypertensive Chinese adults: do methylenetetrahydrofolate reductase and methionine synthase gene polymorphisms affect therapeutic responses? Pharmacogenet Genomics. (2012) 22:421–8. 10.1097/FPC.0b013e32834ac5e8 21869730

[5] Kim YD Jung YH Saposnik G. Traditional risk factors for stroke in East Asia. J Stroke. (2016) 18:273–85. 10.5853/jos.2016.00885 27733028 PMC5066436

[6] Harmey D Hessle L Narisawa S Johnson KA Terkeltaub R Millan JL. Concerted regulation of inorganic pyrophosphate and osteopontin by akp2, enpp1, and ank: an integrated model of the pathogenesis of mineralization disorders. Am J Pathol. (2004) 164:1199–209. 10.1016/S0002-9440(10)63208-7 15039209 PMC1615351

[7] Kunutsor SK Apekey TA Khan H. Liver enzymes and risk of cardiovascular disease in the general population: a meta-analysis of prospective cohort studies. Atherosclerosis. (2014) 236:7–17. 10.1016/j.atherosclerosis.2014.06.006 24998934

[8] Kabootari M Raee MR Akbarpour S Asgari S Azizi F Hadaegh F. Serum alkaline phosphatase and the risk of coronary heart disease, stroke and all-cause mortality: tehran lipid and glucose study. BMJ Open. (2018) 8:e23735. 10.1136/bmjopen-2018-023735 30478120 PMC6254490

[9] Wannamethee SG Sattar N Papcosta O Lennon L Whincup PH. Alkaline phosphatase, serum phosphate, and incident cardiovascular disease and total mortality in older men. Arterioscler Thromb Vasc Biol. (2013) 33:1070–6. 10.1161/ATVBAHA.112.300826 23430618

[10] Xiangyu P Zhao J Wu Y. Serum alkaline phosphatase level is correlated with the incidence of cerebral small vessel disease. Clin Invest Med. (2019) 42:E47–52. 10.25011/cim.v42i1.32392 30904036

[11] Lee HB Kim J Kim SH Kim S Kim OJ Oh SH. Association between serum alkaline phosphatase level and cerebral small vessel disease. PLoS ONE. (2015) 10:e143355. 10.1371/journal.pone.0143355 26580067 PMC4651565

[12] Ryu WS Lee SH Kim CK Kim BJ Kwon HM Yoon BW. High serum alkaline phosphatase in relation to cerebral small vessel disease. Atherosclerosis. (2014) 232:313–8. 10.1016/j.atherosclerosis.2013.11.047 24468144

[13] Shimizu Y Imano H Ohira T Kitamura A Kiyama M Okada T. Alkaline phosphatase and risk of stroke among Japanese: the circulatory risk in communities study (CIRCS). J Stroke Cerebrovasc Dis. (2013) 22:1046–55. 10.1016/j.jstrokecerebrovasdis.2012.06.009 22841505

[14] Men X Sun W Fan F Zhao M Huang X Wang Y. China stroke primary prevention trial: visit-to-visit systolic blood pressure variability is an independent predictor of primary stroke in hypertensive patients. J Am Heart Assoc. (2017) 6:e004350. 10.1161/JAHA.116.004350 28288974 PMC5523997

[15] Qin X Li Y Sun N He M Tang G Yin D. Impact of achieved blood pressure on first stroke in uncomplicated grade 1 hypertension. J Am Heart Assoc. (2017). 6:e005247. 10.1161/JAHA.116.005247 28275067 PMC5524030

[16] Huo Y Li J Qin X Huang Y Wang X Gottesman RF. Efficacy of folic acid therapy in primary prevention of stroke among adults with hypertension in China: the CSPPT randomized clinical trial. JAMA. (2015) 313:1325–35. 10.1001/jama.2015.2274 25771069

[17] Kong X Huang X Zhao M Xu B Xu R Song Y. Platelet count affects efficacy of folic acid in preventing first stroke. J Am Coll Cardiol. (2018) 71:2136–46. 10.1016/j.jacc.2018.02.072 29747834

[18] He M Qin X Cui Y Cai Y Sun L Xu X. Prevalence of unrecognized lower extremity peripheral arterial disease and the associated factors in Chinese hypertensive adults. Am J Cardiol. (2012) 110:1692–8. 10.1016/j.amjcard.2012.07.038 23146365

[19] Qin X Li Y He M Tang G Yin D Liang M. Folic acid therapy reduces serum uric acid in hypertensive patients: a substudy of the China stroke primary prevention trial (CSPPT). Am J Clin Nutr. (2017) 105:882–9. 10.3945/ajcn.116.143131 28148501

[20] Sharma U Pal D Prasad R. Alkaline phosphatase: an overview. Indian J Clin Biochem. (2014) 29:269–78. 10.1007/s12291-013-0408-y 24966474 PMC4062654

[21] Orita Y Yamamoto H Kohno N Sugihara M Honda H Kawamata S. Role of osteoprotegerin in arterial calcification: development of new animal model. Arterioscler Thromb Vasc Biol. (2007) 27:2058–64. 10.1161/ATVBAHA.107.147868 17615383

[22] Takakura N Watanabe T Suenobu S Yamada Y Noda T Ito Y. A role for hematopoietic stem cells in promoting angiogenesis. Cell. (2000) 102:199–209. 10.1016/S0092-8674(00)00025-8 10943840

[23] Yamada Y Takakura N. Physiological pathway of differentiation of hematopoietic stem cell population into mural cells. J Exp Med. (2006) 203:1055–65. 10.1084/jem.20050373 16606664 PMC2118268

[24] Lumachi F Ermani M Camozzi V Tombolan V Luisetto G. Changes of bone formation markers osteocalcin and bone-specific alkaline phosphatase in postmenopausal women with osteoporosis. Ann N Y Acad Sci. (2009) 1173(Suppl 1): E60–3. 10.1111/j.1749-6632.2009.04953.x 19751416

[25] Calvi LM Adams GB Weibrecht KW Weber JM Olson DP Knight MC. Osteoblastic cells regulate the haematopoietic stem cell niche. Nature. (2003) 425:841–6. 10.1038/nature02040 14574413

[26] Zhang J Niu C Ye L Huang H He X Tong WG. Identification of the haematopoietic stem cell niche and control of the niche size. Nature. (2003) 425:836–41. 10.1038/nature02041 14574412

[27] Hager S Lampert FM Orimo H Stark GB Finkenzeller G. Up-regulation of alkaline phospatase expression in human primary osteoblasts by cocultivation with primary endothelial cells is mediated by p38 mitogen-activated protein kinasedependent mRNA stabilization. Tissue Eng Part A. (2009) 15:3437–47. 10.1089/ten.tea.2009.0133 19409035

[28] Perticone F Perticone M Maio R Sciacqua A Andreucci M Tripepi G. Serum alkaline phosphatase negatively affects endothelium-dependent vasodilation in naive hypertensive patients. Hypertension. (2015) 66:874–80. 10.1161/HYPERTENSIONAHA.115.06117 26324506

[29] Schultz-Hector S Balz K Bohm M Ikehara Y Rieke L. Cellular localization of endothelial alkaline phosphatase reaction product and enzyme protein in the myocardium. J Histochem Cytochem. (1993) 41:1813–21. 10.1177/41.12.8245430 8245430

[30] Boo YC Jo H. Flow-dependent regulation of endothelial nitric oxide synthase: role of protein kinases. Am J Physiol Cell Physiol. (2003) 285:C499–508. 10.1152/ajpcell.00122.2003 12900384

