# Supplementary material for: Positive Association Between Serum Alkaline Phosphatase and First Stroke in Hypertensive Adults
Source: Front Cardiovasc Med. 2021 Dec 10;8:749196. doi: 10.3389/fcvm.2021.749196 (PMC8702620; doi:10.3389/fcvm.2021.749196)
Supplement: Supplementary file 1 [file Data_Sheet_1.docx]

**Positive association between serum alkaline phosphatase and first stroke in hypertensive adults**

**Supplemental Figure 1.** Flow chart of the participants

**Supplemental Table 1.** Concomitant medication usage during the treatment period by baseline serum alkaline phosphatase quartiles

**Supplemental Table 2.** The association between baseline alkaline phosphatase and other covariates

**Supplemental Table 3.** The association between baseline alkaline phosphatase and the risk of first stroke in normal ALP levels (20-140 IU/L)

**Supplemental Table 4.** The association between baseline alkaline phosphatase and the risk of first stroke, with further adjustment for the use of calcium channel blockers, diuretics and glucose-lowering drugs during the treatment period

**Supplemental Table 5.** The association between baseline alkaline phosphatase and the risk of first stroke, with further adjustment for AST, ALT, GGT

**Supplemental Table 6.** Serum alkaline phosphatase levels at baseline and after treatment

**Supplemental Table 7.** The association between treatment group and the risk of first total stroke stratified by alkaline phosphatase levels

20,702 participants in CSPPT

Excluded n=126

Missing alkaline phosphatase measurements at baseline

20,576 participants in this analysis

Excluded n=829

Self-reported history of liver disease at baseline

19,747 participants in final analysis

None first stroke

n=19,141 (96.9%)

Fist stroke

n=606 (3.1%)

**Supplemental Figure 1. Flow chart of the participants**

**Supplemental Table 1. Concomitant medication usage during the treatment period by baseline serum alkaline phosphatase quartiles^*^**

| Variables | Baseline serum alkaline phosphatase quartiles, IU/L | | | | *P* value |
| --- | --- | --- | --- | --- | --- |
|  | Q1 (<79) | Q2 (79-<96) | Q3 (96-<118) | Q4 (≥118) |  |
| N | 4695 | 4919 | 5182 | 4951 |  |
| Antihypertensive drugs |  |  |  |  |  |
| Calcium channel blockers | 3665 (78.1) | 3880 (78.9) | 4138 (79.9) | 3950 (79.8) | 0.096 |
| Diuretics | 2511 (53.5) | 2546 (51.8) | 2576 (49.7) | 2136 (43.1) | <0.001 |
| Glucose-lowering drugs | 44 (0.9) | 66 (1.3) | 90 (1.7) | 103 (2.1) | <0.001 |
| Lipid-lowering drugs | 8 (0.2) | 5 (0.1) | 9 (0.2) | 8 (0.2) | 0.773 |
| Antiplatelet drugs | 40 (0.9) | 40 (0.8) | 38 (0.7) | 24 (0.5) | 0.133 |

^*^Regular concomitant medication usage was defined as 180 or more cumulative days of taking the drug of interest.

**Supplemental Table 2. The association between baseline alkaline phosphatase and other covariates**

| Variables | β | *P* value |
| --- | --- | --- |
| Age, year | 0.565 | <0.001 |
| Female | 10.020 | <0.001 |
| Body mass index, kg/m^2^ | -0.132 | 0.028 |
| Systolic blood pressure, mmHg | 0.030 | 0.005 |
| Time-averaged on-treatment SBP, mmHg | 0.063 | 0.001 |
| Current smoking | 2.833 | <0.001 |
| Current alcohol drinking | -6.621 | <0.001 |
| *MTHFR* 677 TT | 0.386 | 0.514 |
| Enalapril-folic acid | -0.125 | 0.751 |
| Antihypertensive drugs | -3.420 | <0.001 |
| **Laboratory results** |  |  |
| Total cholesterol, mmol/L | -1.025 | <0.001 |
| Triglycerides, mmol/L | 1.045 | <0.001 |
| Fasting glucose, mmol/L | 1.619 | <0.001 |
| Creatinine, μmol/L | -0.060 | <0.001 |
| Total homocysteine, μmol/L | 0.124 | <0.001 |
| Albumin, g/L | 1.167 | <0.001 |

**Supplemental Table 3. The association between baseline alkaline phosphatase and the risk of first stroke in normal ALP levels (20-140 IU/L)**

| ALP, IU/L | N | No. of events (%) | Crude model | |  | Adjusted model^*^ | |
| --- | --- | --- | --- | --- | --- | --- | --- |
|  |  |  | HR (95% CI) | *P* value |  | HR (95% CI) | *P* value |
| **First total stroke** | |  |  |  |  |  |  |
| Continuous, per SD increment | 17725 | 543 (3.1) | 1.09 (1.00, 1.18) | 0.056 |  | 1.15 (1.05, 1.27) | 0.003 |
| Quartiles |  |  |  |  |  |  |  |
| Q1 (<78) | 4401 | 112 (2.5) | 1.00 |  |  | 1.00 |  |
| Q2 (78-<93) | 4376 | 141 (3.2) | 1.28 (1.00, 1.63) | 0.055 |  | 1.34 (1.03, 1.73) | 0.028 |
| Q3 (93-<110) | 4406 | 144 (3.3) | 1.28 (1.00, 1.64) | 0.048 |  | 1.42 (1.09, 1.84) | 0.009 |
| Q4 (≥110) | 4542 | 146 (3.2) | 1.27 (0.99, 1.62) | 0.061 |  | 1.47 (1.12, 1.93) | 0.005 |
| *P* for trend |  |  | 0.083 |  |  | 0.007 |  |
| Categories |  |  |  |  |  |  |  |
| Q1 (<78) | 4401 | 112 (2.5) | 1.00 |  |  | 1.00 |  |
| Q2-4 (≥78) | 13324 | 431 (3.2) | 1.27 (1.04, 1.57) | 0.022 |  | 1.40 (1.12, 1.75) | 0.003 |
| **First ischemic stroke** | |  |  |  |  |  |  |
| Continuous, per SD increment | 17725 | 442 (2.5) | 1.09 (0.99, 1.20) | 0.067 |  | 1.17 (1.06, 1.30) | 0.003 |
| Categories |  |  |  |  |  |  |  |
| Q1 (<78) | 4401 | 93 (2.1) | 1.00 |  |  | 1.00 |  |
| Q2-4 (≥78) | 13324 | 349 (2.6) | 1.24 (0.99, 1.56) | 0.064 |  | 1.35 (1.05, 1.72) | 0.017 |
| **First hemorrhagic stroke** | | |  |  |  |  |  |
| Continuous, per SD increment | 17725 | 99 (0.6) | 1.07 (0.88, 1.30) | 0.527 |  | 1.10 (0.88, 1.37) | 0.426 |
| Categories |  |  |  |  |  |  |  |
| Q1 (<78) | 4401 | 18 (0.4) | 1.00 |  |  | 1.00 |  |
| Q2-4 (≥78) | 13324 | 81 (0.6) | 1.48 (0.89, 2.47) | 0.130 |  | 1.85 (1.02, 3.34) | 0.042 |

^*^Adjusted for study centers, treatment groups, age, sex, body mass index, smoking, alcohol drinking, systolic blood pressure (SBP), albumin, total cholesterol, triglyceride, fasting glucose, creatinine, total homocysteine, methylenetetrahydrofolate reductase (*MTHFR*) C677T genotypes and antihypertensive treatment at baseline, as well as time-averaged SBP during the treatment period.

**Abbreviations:** ALP, alkaline phosphatase.

**Supplemental Table 4. The association between baseline alkaline phosphatase and the risk of first stroke, with further adjustment for** **the use of** **calcium channel blockers, diuretics and glucose-lowering drugs during the treatment period**

| ALP, IU/L | N | No. of events (%) | Crude model | |  | Adjusted model^*^ | |
| --- | --- | --- | --- | --- | --- | --- | --- |
|  |  |  | HR (95% CI) | *P* value |  | HR (95% CI) | *P* value |
| **First total stroke** | |  |  |  |  |  |  |
| Categories |  |  |  |  |  |  |  |
| Q1 (<79) | 4695 | 123 (2.6) | 1.00 |  |  | 1.00 |  |
| Q2-4 (≥79) | 15052 | 483 (3.2) | 1.22 (1.00, 1.49) | 0.046 |  | 1.38 (1.12, 1.72) | 0.003 |
| **First ischemic stroke** | |  |  |  |  |  |  |
| Categories |  |  |  |  |  |  |  |
| Q1 (<79) | 4695 | 101 (2.2) | 1.00 |  |  | 1.00 |  |
| Q2-4 (≥79) | 15052 | 392 (2.6) | 1.21 (0.97, 1.51) | 0.088 |  | 1.37 (1.08, 1.73) | 0.010 |
| **First hemorrhagic stroke** | |  |  |  |  |  |  |
| Categories |  |  |  |  |  |  |  |
| Q1 (<79) | 4695 | 21 (0.4) | 1.00 |  |  | 1.00 |  |
| Q2-4 (≥79) | 15052 | 90 (0.6) | 1.33 (0.83, 2.14) | 0.239 |  | 1.65 (0.95, 2.85) | 0.074 |

^*^Adjusted for study centers, treatment groups, age, sex, body mass index, smoking, alcohol drinking, systolic blood pressure (SBP), albumin, total cholesterol, triglyceride, fasting glucose, creatinine, total homocysteine, methylenetetrahydrofolate reductase (*MTHFR*) C677T genotypes and antihypertensive treatment at baseline, as well as time-averaged SBP, the use of calcium channel blockers, diuretics and glucose-lowering during the treatment period.

**Abbreviations:** ALP, alkaline phosphatase.

**Supplemental Table 5. The association between baseline alkaline phosphatase and the risk of first stroke, with further adjustment for AST, ALT, GGT**

| ALP, IU/L | N | No. of events (%) | Crude model | |  | Adjusted model^*^ | |
| --- | --- | --- | --- | --- | --- | --- | --- |
|  |  |  | HR (95% CI) | *P* value |  | HR (95% CI) | *P* value |
| **First total stroke** | |  |  |  |  |  |  |
| Categories |  |  |  |  |  |  |  |
| Q1 (<79) | 4695 | 123 (2.6) | 1.00 |  |  | 1.00 |  |
| Q2-4 (≥79) | 15052 | 483 (3.2) | 1.22 (1.00, 1.49) | 0.046 |  | 1.40 (1.12, 1.73) | 0.002 |
| **First ischemic stroke** | |  |  |  |  |  |  |
| Categories |  |  |  |  |  |  |  |
| Q1 (<79) | 4695 | 101 (2.2) | 1.00 |  |  | 1.00 |  |
| Q2-4 (≥79) | 15052 | 392 (2.6) | 1.21 (0.97, 1.51) | 0.088 |  | 1.38 (1.09, 1.75) | 0.008 |
| **First hemorrhagic stroke** | |  |  |  |  |  |  |
| Categories |  |  |  |  |  |  |  |
| Q1 (<79) | 4695 | 21 (0.4) | 1.00 |  |  | 1.00 |  |
| Q2-4 (≥79) | 15052 | 90 (0.6) | 1.33 (0.83, 2.14) | 0.239 |  | 1.60 (0.93, 2.77) | 0.091 |

**^*^**Adjusted for study centers, treatment groups, age, sex, body mass index, smoking, alcohol drinking, systolic blood pressure (SBP), albumin, total cholesterol, triglyceride, fasting glucose, creatinine, total homocysteine, methylenetetrahydrofolate reductase (*MTHFR*) C677T genotypes and antihypertensive treatment, AST, ALT, GGT at baseline, as well as time-averaged SBP during the treatment period.

**Abbreviations:** ALP, alkaline phosphatase; ALT, alanine aminotransferase; AST, aspartate aminotransferase; GGT, gamma glutamyl transpeptidase.

**Supplemental Table 6. Serum alkaline phosphatase levels at baseline and after treatment**

| Variables ^a^ | Treatment group | | *P* value |
| --- | --- | --- | --- |
|  | Enalapril-only | Enalapril-folic acid |  |
| N | 9864 | 9883 |  |
| ALP at baseline, IU/L | 100.7 ± 31.1 | 100.8 ± 31.2 | 0.818 |
| ALP at the exit, IU/L | 92.7 ± 34.4 | 92.5 ± 29.1 | 0.744 |
| Change in ALP, IU/L | -7.7 ± 30.2 | -7.8 ± 24.4 | 0.669 |

^a^ The variables are presented as Mean ± SD

**Abbreviations:** ALP, alkaline phosphatase.

**Supplemental Table 7. The association between treatment group and the risk of first total stroke stratified by alkaline phosphatase levels**

| ALP, IU/L | N | No. of events (%) | Crude model | | Adjusted model ^a^ | | *P*-interaction |
| --- | --- | --- | --- | --- | --- | --- | --- |
|  |  |  | HR (95% CI) | *P* value | HR (95% CI) | *P* value |  |
| **ALP <79 IU/L** |  |  |  |  |  |  | 0.597 |
| Enalapril-only group | 2320 | 66 (2.8) | 1.00 |  | 1.00 |  |  |
| Enalapril-folic acid group | 2375 | 57 (2.4) | 0.84 (0.59,1.20) | 0.331 | 0.89 (0.61, 1.29) | 0.537 |  |
| **ALP ≥79 IU/L** |  |  |  |  |  |  |  |
| Enalapril-only group | 7544 | 272 (3.6) | 1.00 |  | 1.00 |  |  |
| Enalapril-folic acid group | 7508 | 211 (2.8) | 0.78 (0.65, 0.93) | 0.006 | 0.78 (0.65, 0.94) | 0.007 |  |

^a^ Adjusted for study centers, age, sex, body mass index, smoking, alcohol drinking, systolic blood pressure (SBP), albumin, total cholesterol, triglyceride, fasting glucose, creatinine, total homocysteine, methylenetetrahydrofolate reductase (*MTHFR*) C677T genotypes and antihypertensive treatment at baseline, as well as time-averaged SBP during the treatment period.

**Abbreviations:** ALP, alkaline phosphatase.
